# Supplementary material for: The interactome of intact mitochondria by cross-linking mass spectrometry provides evidence for coexisting respiratory supercomplexes
Source: Mol Cell Proteomics. 2017 Dec 8;17(2):216–32. doi: 10.1074/mcp.RA117.000470 (PMC5795388; doi:10.1074/mcp.RA117.000470)
Supplement: Supplemental Data [file supp_RA117.000470_133922_0_supp_23978_fzffwf.zip › spectra_annotation/mito_DR_spectra_annotation/359-1-5-1-1-1.pdf]

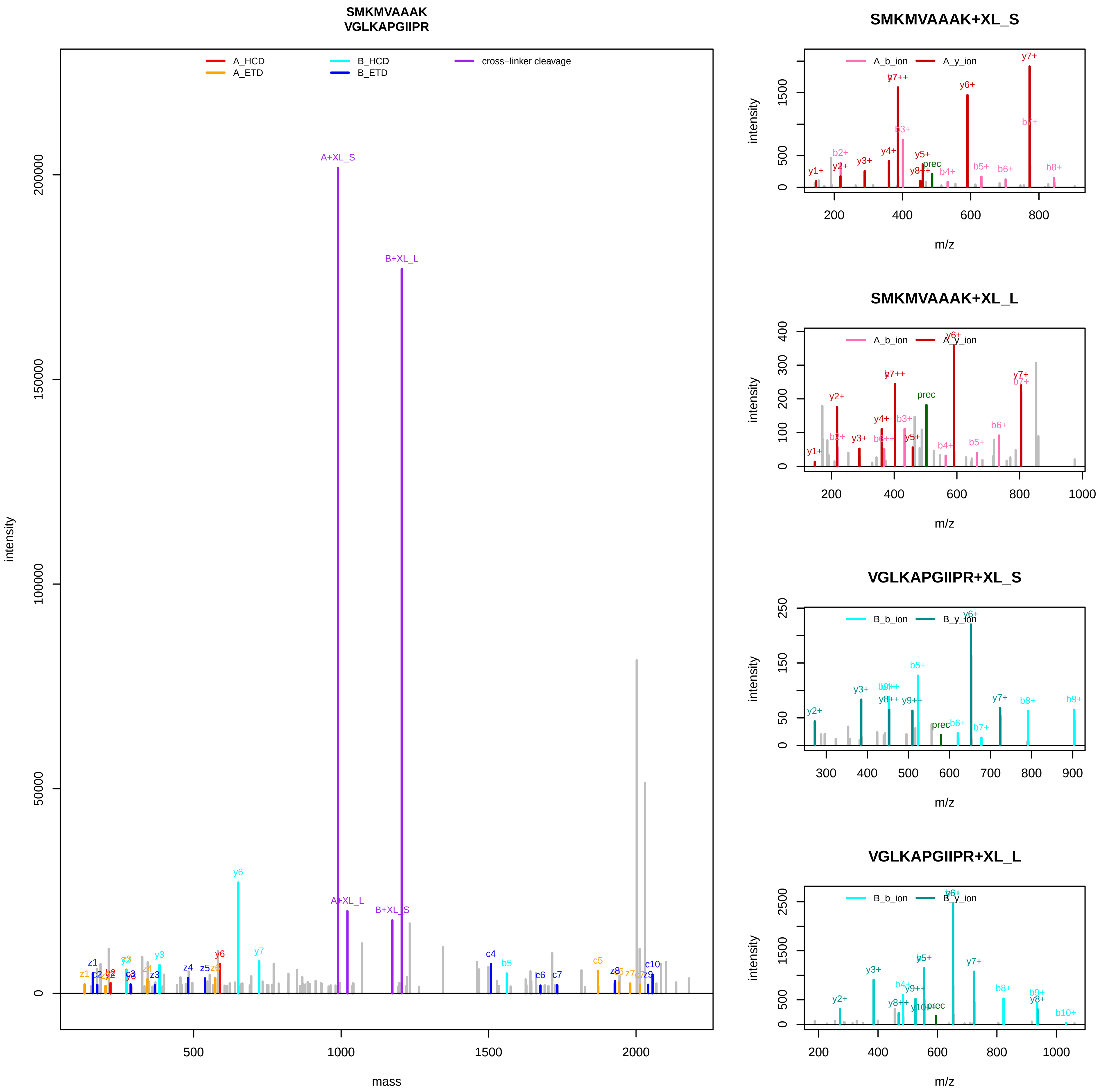
SMKMVAAAK
VGLKAPGIIPR
A_HCD
A_ETD
B_HCD
B_ETD
cross−linker cleavage
A+XL_S
B+XL_L
A+XL_L
B+XL_S
intensity
mass
0
50000
100000
150000
200000
500
1000
1500
2000
y6
y7
y3
c4
b5
c5
c6
c7
z8
c10
z9
z1
z3
z4
z5
y6
SMKMVAAAK+XL_S
A_b_ion
A_y_ion
intensity
m/z
y7+
y7++
y6+
b7+
b3+
y4+
y5+
prec
y3+
b2+
y2+
y1+
y8++
b4+
b5+
b6+
b8+
SMKMVAAAK+XL_L
A_b_ion
A_y_ion
intensity
m/z
y6+
y7++
y7+
b7+
prec
y2+
y4+
b3+
y3+
y5+
b6+
b5+
b4+
y1+
VGLKAPGIIPR+XL_S
B_b_ion
B_y_ion
intensity
m/z
y6+
b5+
y3+
y8++
y9++
y7+
b8+
b9+
y2+
prec
b6+
b7+
VGLKAPGIIPR+XL_L
B_b_ion
B_y_ion
intensity
m/z
y6+
y5+
y3+
y7+
b4+
y9++
b8+
b9+
y8+
y2+
y8++
prec
b10+
